# Stiffening of sickle cell trait red blood cells under simulated strenuous exercise conditions

**DOI:** 10.1038/micronano.2016.61

**Published:** 2016-11-07

**Authors:** Zhensong Xu, Yi Zheng, Xian Wang, Nadine Shehata, Chen Wang, Shaorong Xie, Yu Sun

**Affiliations:** 1 Department of Mechanical and Industrial Engineering, University of Toronto, Toronto, ON, Canada; 2 Institute of Biomaterials and Biomedical Engineering, University of Toronto, Toronto, ON, Canada; 3 Department of Medicine, University of Toronto, Toronto, ON, Canada; 4 Department of Pathology and Laboratory Medicine, Mount Sinai Hospital, Toronto, ON, Canada; 5 Department of Laboratory Medicine and Pathobiology, University of Toronto, Toronto, ON, Canada; 6 School of Mechatronic Engineering and Automation, Shanghai University, Shanghai, China; 7 Department of Electrical and Computer Engineering, University of Toronto, Toronto, ON, Canada

**Keywords:** deformability, microfluidics, shear modulus, sickle cell trait, stiffening, red blood cell

## Abstract

The higher risk of vaso-occlusion events and sudden death for sickle-cell trait (SCT) athletes has been speculatively ascribed to SCT red blood cell (RBC) stiffening during strenuous exercise. However, the microenvironmental changes that could induce the stiffening of SCT RBCs are unknown. To address this question, we measured the mechanical properties of and changes in SCT RBCs under deoxygenated and acidic environments, which are two typical conditions present in the circulation of athletes undertaking strenuous exercise. The results reveal that SCT RBCs are inherently stiffer than RBCs from non-SCT healthy subjects, and a lower pH further stiffens the SCT cells. Furthermore, at both normal and low pH levels, deoxygenation was found to not be the cause of the stiffness of SCT RBCs. This study confirms that the stiffening of SCT RBCs occurs at a low pH and implies that SCT RBC stiffening could be responsible for vaso-occlusion in SCT athletes during strenuous exercise.

## Introduction

Athletes are often considered the healthiest members of the human population; hence, the occurrence of sudden deaths in this group^1^ can be shocking and has a devastating impact on communities and the public^2,3^. According to an analysis conducted by the National Collegiate Athletic Association (NCAA) from 2004 to 2008 (Ref. 1), 36 out of 80 medical causes of athletes’ death (45%) were identified to be exertional sudden deaths^4^. Among all exertional sudden deaths, sickle cell trait (SCT)-related cases caused the most controversies^5–7^.

SCT describes the inheritance of one normal hemoglobin gene (*HbA*) from one parent along with one mutated β1-globin gene, the sickle hemoglobin gene (*HbS*), from the other parent^8^. At the end of 2009, there were ~300 million people worldwide with SCT.

SCT is typically considered to be benign and harmless^9^. However, as more cases of SCT athletes’ sudden deaths were reported^4,10^, heated debates over whether SCT should be considered as a cause of death during exercise and whether athletes should be screened for SCT arose. Rationally, analyzing these issues requires a clear understanding of the properties of SCT RBCs^4,11,12^.

Vaso-occlusion, usually caused by the blockage of blood vessels, is one of the most fatal and common symptoms of sickle cell disease (SCD). It also appears to be a crucial contributor to sudden deaths in SCT individuals^12^. RBCs from SCD patients are known to become stiffer and even sickled under deoxygenated or acidic conditions, causing the blockage of blood vessels^13,14^. However, whether SCT RBCs also become stiffer under deoxygenated or acidic conditions is not known.

During strenuous exercise, athletes’ muscles are under a maximal oxygen consuming condition, which lowers the oxygen level in the circulation. Furthermore, although human blood pH is normally 7.35, in strenuous exercise, a higher concentration of hydrogen ions in the human body causes blood pH to drop below 7.0 and even to 6.8 under extreme conditions for a short time period^15,16^.

In this study, we focused on determining whether lowered pH and deoxygenation conditions can trigger the stiffening of SCT RBCs, which could be associated with higher vaso-occlusion risks. Measurements were taken on both normal RBCs and SCT RBCs using a microfluidic system that is capable of controlling oxygen and pH levels.

## Materials and methods

### Blood specimens

The study was performed in accordance with the institutional guidelines for using human tissue samples. Blood samples were collected for routine tests and used for study only after they were completed for clinical tests and would be otherwise discarded. The study protocol was approved by the Mount Sinai Hospital Research Ethics Board, in which informed consent was not required because the samples were selected retrospectively, no patient identification was disclosed to the study, and the study had no effect on the clinical test or patient management. SCT was confirmed by sickle cell testing and standard hemoglobin electrophoresis in the clinical laboratory. Blood samples, including seven normal blood samples and seven SCT blood samples, were stored with ethylenediaminetetraacetic acid (EDTA, 1.5 mg ml^−1^) and used within 48 h. Before they were introduced into the device under room temperature, blood samples tested under normal pH level were diluted 200 times in phosphate-buffered saline (PBS, pH=7.35±0.05), whereas blood samples tested under acidic conditions were diluted 200 times in acid-adjusted PBS (pH=7.10±0.05 and pH=6.85±0.05).

### Device and measurement

**Figure 1:**
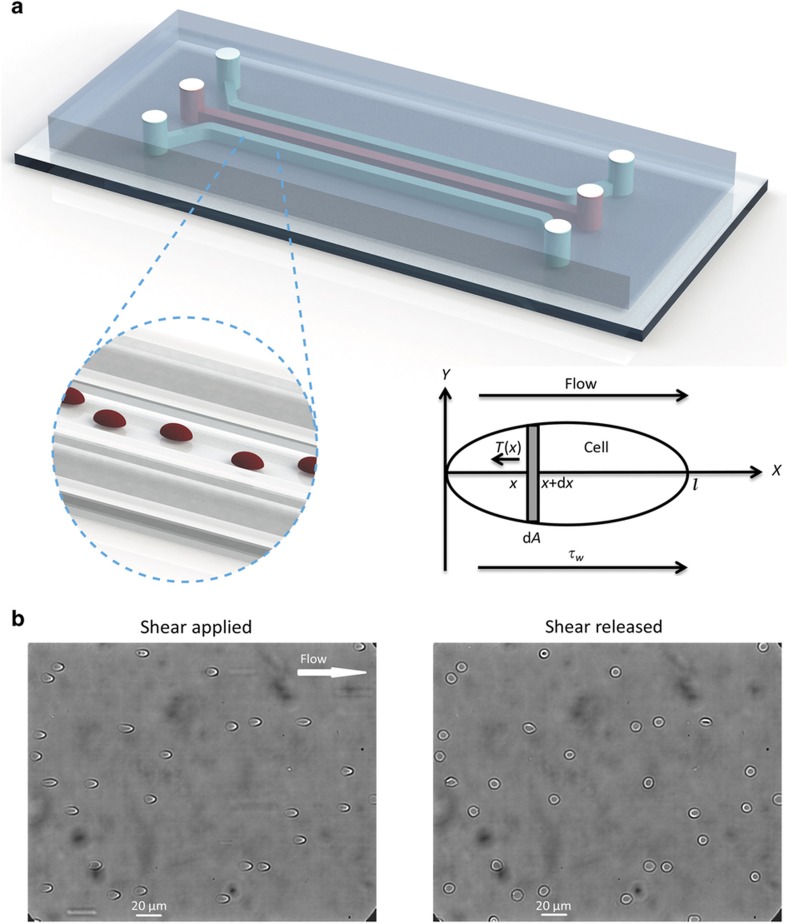
(**a**) Schematic of the microfluidic device used for RBC mechanical property measurement. RBCs in solutions of different pH levels are loaded into the middle channel and adhere onto the glass channel bottom. The oxygen level is accurately varied by pumping air or nitrogen into the two side channels. Schematic illustration of the deformation of a cell element. (**b**) RBCs are deformed under shear stress (1.8 kPa). After the release of shear stress, RBCs recover to their original shape.

As shown in Figure 1a, the microfluidic device consists of three parallel channels. The middle channel is used for loading and testing RBCs, and the other two channels are used to control the oxygen level in the middle channel. After a diluted blood sample is introduced into the middle channel, RBCs strongly adhere to the glass substrate due to the difference in electrical charge on the cell membrane and glass surface^17^. In experiments, when the pressure was varied from 0 Pa to 9 kPa, none of the RBCs were detached or revealed noticeable displacements. Two water tanks containing PBS are connected to the inlet and outlet of the middle channel to maintain the osmolality and pH level. Pumping either air or nitrogen into the two side channels controls the oxygen level in the device owing to the gas permeability characteristic of polydimethylsiloxane (PDMS)^18^. The cross-sectional area of the three channels is 60 μm×300 μm. The gap between neighboring channels is 100 μm for facilitated gas exchange between the gas channel and the cell testing channel. Details of fabrication and design of the microfluidic device have been described in our previous study^19^. In addition, the pH level is controlled by adding hydrochloric acid and confirmed before and after each experiment by using a pH measurement instrument (Hanna FC 240B pH electrode). RBCs are deformed under shear stress (0.9 Pa) generated by a regulated vacuum source (pressure difference is 1.8 kPa), and this shear stress is comparable to the *in vivo* condition^20^. After the release of the shear stress, the RBCs recover to their original shapes. The dynamic recovery process is captured using a CCD camera connected to a microscope. Mechanical models are developed to extract the shear modulus of each RBC.

## Results

### Determination of RBC shear modulus

When an RBC is in a shear flow, its membrane undergoes deformation by shear stress. According to the Kelvin–Voigt (KV) model^21,22^,

(1)T¯=µ2(λ2−1λ2)

where $\bar{T}$ (μN m^−1^) is the average tension force acting on the RBC membrane, *μ* (μN m^−1^) is the elastic shear modulus expressed in force per unit length, and *λ* is the extension ratio of the RBC membrane. $\lambda =\frac{l}{l_{0}}$, where *l* is the RBC’s length when deformed under shear stress, and *l*_0_ is the RBC’s original length.

The flow in the microfluidic channel in this study is driven by a pressure difference (Δ*P*). The velocity profile of pressure-driven flow is

(2)ν=πR24ηΔPL(1−r2R2)

where *η* is the dynamic viscosity of the fluid and *L* is the length of the channel. Because the microchannel has a rectangular cross-section and its width (*w*) is much larger than the channel height (*h*), hydraulic radius *R* is approximately equal to *h*. Because *w*>>*h*, the error caused by the approximation is minor and does not affect the result in this paper. Shear stress on the microchannel bottom where RBCs are located is

(3)τw=ηdνdr|r=R=ΔPh2L

To calculate tension force $\bar{T}$ in the direction of extension, a small element (d*A*) is taken for force equilibrium analysis, as shown in Figure 1, and shear flow only applies in the *x* direction. Because only steady-state behavior is considered, the tension force and shear force on each element are balanced^21,23^.

(4)2d[TxY(x)]=τwdA

where *Y*(*x*) is the half-width of the element d*A*. The average tension force $\bar{T}$ is^24^

(5)T¯=∫0lT(x)dxl≈τwAlb

where *A* is the surface area of the RBC, and *l*_b_ is the width of the RBC. Shear modulus *μ* is determined by substituting Equation (5) into Equation (1).

### SCT RBCs become stiffened under lower pH

**Figure 2:**
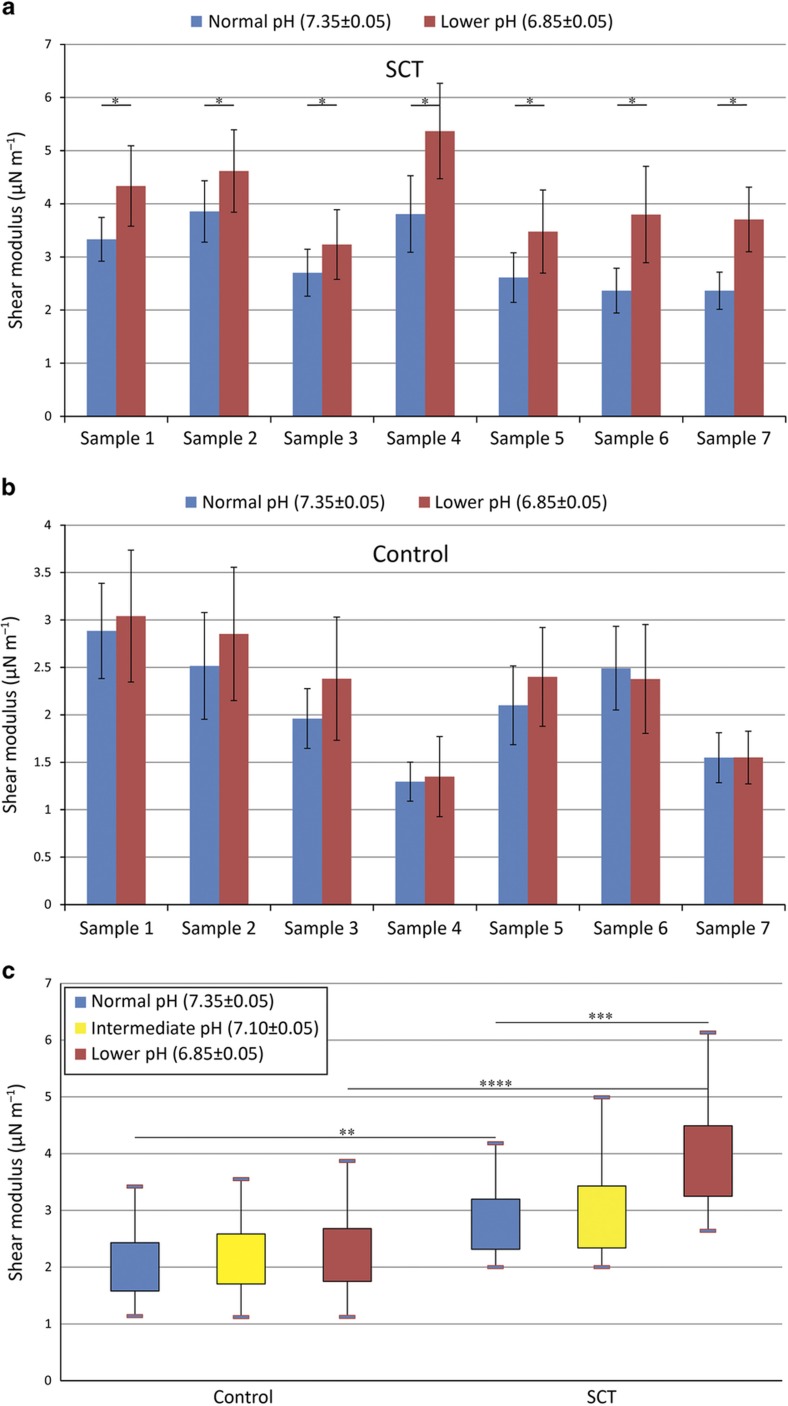
(**a**) Shear modulus of SCT RBCs under a physiological pH level of 7.35 (blue) and an acidic pH level of 6.85 (red). Error bars represent the standard deviation. All samples (*n*=30–70 for each sample) show a significant difference between different pH levels (*P<0.05). (**b**) The shear modulus of normal RBCs did not reveal a significant difference under different pH levels (*P* value>0.05; *n*=30–70 for each sample). Error bars represent the standard deviation. (**c**) Boxplot showing the summarized shear modulus of RBCs from the seven tested SCT samples and the seven tested normal samples under different pH conditions (**P=5.5×10^−15^, ***P=2.2×10^−29^, ****P=1.9×10^−82^; *n*=200–300).

RBCs from healthy donors (control) and SCT individuals were first tested using the microsystem without adjusting the pH level. Figure 2 shows that under normal pH levels, SCT RBCs are significantly stiffer than normal RBCs, which agrees with previous results^19,25^. The higher stiffness of SCT RBCs could lead to a higher blood viscosity in the vascular system^26^. In large blood vessels, lower RBC deformability limits cell orientation in flow and thus increases blood viscosity^27^. In small blood vessels, stiffer RBCs lead to a lower Fahraeus–Lindqvist effect, which increases the flow resistance and blood viscosity^28^. In healthy non-SCT carriers, endothelial cells lining the blood vessels can generate vasodilators (e.g., nitric oxide) to mediate increased blood viscosity. In contrast, SCT carriers are known to develop impaired vascular functions and generate fewer vasodilators^12,29^.

**Figure 3:**
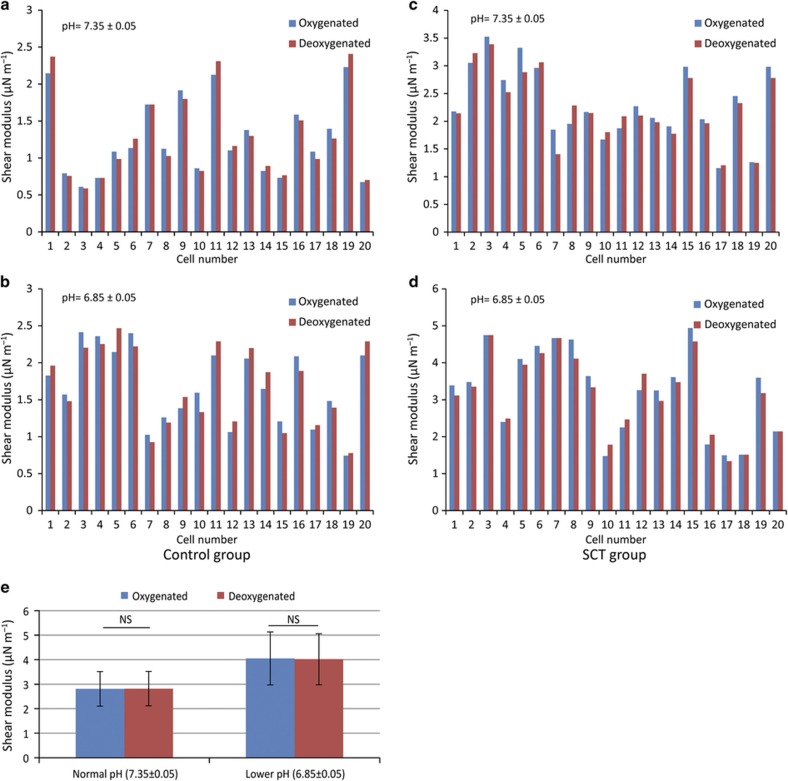
At physiological pH 7.35 (**a**) and low pH 6.85 (**b**), the normal RBCs’ elastic modulus did not change significantly when the channel was deoxygenated (*P>0.75). (**c** and **d**) For SCT RBCs, although the shear modulus increased at pH 6.85 compared with pH 7.35, deoxygenation did not induce a further increase (*P>0.75). (**e**) Summarized shear modulus of SCT RBCs under different oxygen levels, in which no difference was observed. Error bars represent the standard deviation.

We next measured the shear modulus of both SCT and normal RBCs at lower pH levels. As shown in Figure 2a, the shear modulus of all seven tested SCT RBC samples consistently increased under a low pH level of 6.85 (*P*<0.05). This low pH level was chosen because it is known that in strenuous exercise, a higher concentration of hydrogen ions in the human body can cause the pH level to drop to approximately 6.8 (Refs. 15,^16^). We then aimed to investigate whether moderate exercise could induce SCT RBC stiffening. Hence, we conducted experiments under an intermediate pH value (pH 7.10) that mimics the moderate exercise condition, and no significant difference in shear modulus was observed, as shown in Figure 3c.

In contrast, RBCs from normal subjects did not respond significantly to low pH levels. When pH was reduced to 6.85, as shown in Figure 2b including seven samples’ data, the shear modulus of normal RBCs increased slightly. However, the difference was not statistically significant (*P*⩾0.05). In our experiments, normal RBCs started to reveal stiffness changes with statistical significance only at pH levels < 6.0 (data not shown), which is a physiologically irrelevant condition^30–32^.

As shown in Figure 2c in which all seven samples are summarized together and reported in boxplots, when pH was reduced from 7.35 to 6.85, SCT RBCs were stiffened significantly, whereas control RBCs’ stiffness increased only slightly. The average shear modulus of normal RBCs increased from 2.09±0.67 to 2.27±0.83 μN m^−1^, whereas the average shear modulus of RBCs from SCT individuals increased significantly from 2.81±0.7 to 4.05±1.08 μN m^−1^. The results indicate that SCT RBCs are inherently stiffer than control/normal RBCs, and SCT RBCs are more sensitive to lowered pH levels than normal RBCs. Statistical analysis confirms a significant difference of the shear moduli of normal RBCs and SCT RBCs under the acidic condition (2.27±0.83 vs. 4.05±1.08 μN m^−1^; *P*=1.9×10^−82^). The significant stiffening of SCT RBCs under the acidic condition could cause difficulties for the SCT RBCs to pass through minuscule vessels and capillaries. Along with the increased blood viscosity, this could lead to a higher chance of vessel blockage. The resulting vaso-occlusion events could result in acute ventricular failure^33–35^, which could contribute to sudden death in SCT carriers.

### Hypoxia does not induce SCT RBC stiffening

We then tested the oxygen effect on SCT and normal RBCs. As shown in Figure 3, the stiffness of SCT RBCs was not found to increase after deoxygenation under physiological pH (7.35), which is in agreement with the previously reported result^19^. We speculated that under acidic conditions, deoxygenation might cause SCT RBCs to become even stiffer compared with the condition of low pH only. However, the measurement results revealed that deoxygenation is not capable of stiffening SCT RBCs further. In the deoxygenation experiments, we infused nitrogen into the two side channels for 20 min to reduce the oxygen concentration from 20% (air) to 0% (pure nitrogen). The validation of oxygen depletion was described in our previous study^19^.

As discussed in the previous section, the shear modulus of SCT RBCs increased from 2.81±0.7 to 4.05±1.08 μN m^−1^ under pH 7.35 and 6.85. These shear modulus values largely remained the same when the channel was deoxygenated (for pH 7.35, oxygenated: 2.81±0.7 μN m^−1^ vs. deoxygenated: 2.82±0.7 μN m^−1^; for pH 6.85, oxygenated: 4.05±1.08 μN m^−1^ vs. deoxygenated: 4.02±1.04 μN m^−1^). The differences were confirmed by using Mann–Whitney nonparametric analysis to be insignificant (*P*>0.75). To decouple the effect of deoxygenation and pH levels, the RBC samples were diluted 200 times, and it was confirmed after each experiment that there was no significant difference in pH after deoxygenation.

## Discussion

SCT was recently reported to be associated with strenuous exercise-related mortality^4,5,10^. Existing results on the mechanical properties of RBCs from SCT individuals are limited. Here we examined SCT RBCs’ stiffness change under controlled oxygen and pH levels. The results reveal that SCT RBCs are significantly stiffer than RBCs from non-SCT healthy subjects. A lower pH resulted in a 28% increase in SCT RBCs’ shear modulus (Figure 2).

The stiffness increase of SCT RBCs could be due to several physiological alterations. It was reported that SCT RBCs contain a higher concentration of Ca^+2^, which can enhance the binding of the cytoplasmic domain of band 3 (CDB3) to the cytoskeleton-bound Ankyrin^25,36^. This stronger binding caused by increased Ca^+2^ can possibly contribute to the higher rigidity of the membrane of SCT RBCs. At lower pH, CDB3 becomes even more compact^30,37^, which can further enhance the binding and stiffening of SCT RBCs. In addition, monocarboxylate transporter 1 (MCT-1) activity has been speculated to impact RBC stiffness^38–40^. Because MCT-1 activity is inherently stronger in SCT RBCs than normal RBCs at low pH, the higher concentration of hydrogen ions leads to even stronger MCT-1 activity, and the enhancement of MCT-1 activity could also be responsible for the significant increase in shear modulus measured on SCT RBCs at pH 6.85. However, because no significant difference was observed when pH was lowered to 7.10, we speculate that at pH 7.10, the cytoplasmic domain of band 3 (CDB3) was not sufficiently compact to enhance the binding to the cytoskeleton-bound Ankyrin, leading to insignificant stiffening of RBCs. The results also suggest that under normal exercise conditions in which pH only slightly decreases, the stiffening of SCT RBCs would not be significantly evident.

The experimental results also show that deoxygenation did not induce further stiffening of SCT RBCs (Figure 3). During strenuous exercise, both blood oxygen and pH levels decreased. Although deoxygenation did not directly impact the stiffness of SCT RBCs, low oxygen levels during exercise can contribute to the lowering of blood pH^41^. Our data measured in the simulated strenuous exercise condition indicate that low pH rather than hypoxia is effective in triggering SCT RBC stiffening. In addition, although SCD RBCs are known to sickle under acidic and/or hypoxia conditions, no sickling of SCT RBCs was observed under these conditions in this study. In addition to the stiffening of SCT RBCs, other exercise-induced physiological changes can also possibly lead to a higher risk. For example, higher epinephrine during exercise has an effect on SCT RBCs’ adhesion^42,43^. Increased adhesion can contribute to stronger interactions between RBCs and epithelial cells and thus trigger inflammatory pathways. Dehydration, a common condition occurring during exercise, has also been reported to affect RBCs’ physical properties^44^. The stiffening of SCT RBCs, inflammation, and dehydration individually and together can be associated with a higher risk among SCT individuals during strenuous exercise.

## Conclusion

This study sought to address the question of whether RBCs in SCT individuals become stiffened during strenuous exercise. RBCs from SCT individuals and non-SCT subjects were tested under simulated strenuous exercise conditions (i.e., low oxygen and low pH). The results show that RBCs from SCT individuals are inherently stiffer and are sensitive to low pH, which induces a significant stiffness increase in SCT RBCs, implying that the stiffening of RBCs could occur in SCT individuals during strenuous exercise. Furthermore, the experimental results revealed that deoxygenation alone did not cause SCT RBCs to increase their stiffness. However, because low oxygen levels contribute to the lowering of blood pH, the stiffening of SCT RBCs *in vivo* could result from a combined effect of low oxygen and low pH.
